# Elemental Composition of Algae-Based Supplements by Energy Dispersive X-ray Fluorescence

**DOI:** 10.3390/plants10102041

**Published:** 2021-09-28

**Authors:** Fernando H. Reboredo, Walter Junior, Maria F. Pessoa, Fernando C. Lidon, José C. Ramalho, Roberta G. Leitão, Maria Manuela Silva, Nuno Alvarenga, Mauro Guerra

**Affiliations:** 1 GeoBioTec, Departamento de Ciências da Terra, Faculdade de Ciências e Tecnologia, Universidade NOVA de Lisboa, Campus da Caparica, 2829-516 Caparica, Portugal; paodequeijo@sapo.pt (W.J.); mfgp@fct.unl.pt (M.F.P.); fjl@fct.unl.pt (F.C.L.); cochichor@mail.telepac.pt (J.C.R.); 2 Plant Stress & Biodiversity Lab, Centro de Estudos Florestais (CEF), Instituto Superior Agronomia (ISA), Universidade de Lisboa (ULisboa), Quinta do Marquês, Av. República, 2784-505 Lisboa, Portugal; 3 LIBPHYS-UNL, Physics Department, NOVA School of Science and Technology, NOVA University Lisbon, 2829-516 Caparica, Portugal; rg.leitao@fct.unl.pt; 4 ESEAG-COFAC, Avenida do Campo Grande 376, 1749-024 Lisboa, Portugal; abreusilva.manuela@gmail.com; 5 Instituto Nacional de Investigação Agrária e Veterinária, I.P., UTI—Unidade de Tecnologia e Inovação, Avenida da República, Quinta do Marquês, 2780-157 Oeiras, Portugal; bartolomeu.alvarenga@ipbeja.pt

**Keywords:** algae, elemental composition, food supplements, risk assessment, X-ray fluorescence

## Abstract

The aim of this study is to evaluate the elemental composition of fifteen algae-based supplements commonly sold in the Portuguese market, by energy dispersive X-ray fluorescence. Despite the fact that the majority of Kelp samples were a good source of iodine, the levels observed might well contribute to an excess in the human body, which can cause dysfunction of the thyroid gland. Furthermore, the presence of lead in Sea spaghetti, Arame, Hijiki and Wakame caused a considerable risk to public health vis a vis possible ingestion of a high daily dose. Regarding arsenic, great variability was observed in all the samples with concentrations equal to or above 60 μg/g in the case of Arame, KelpJ and Hijiki. Although algae mainly accumulate organic arsenic, some also contain high levels of its inorganic form, as is commonly pointed out for Hijiki. Thus, regular ingestion of these supplements must also take into account the mentioned facts. There is no doubt that these supplements are also good sources of other nutrients, but the lack of accurate regulations and control should alert consumers to avoid indiscriminate use of these types of products.

## 1. Introduction

The use of algae as a staple food has a millenary tradition in the East, mainly in Asian countries. In Japan, over 20 species of red, green and brown algae (seaweed) are included in meals [1], with emphasis on the three most popular seaweed products—nori (*Porphyra*), wakame (*Undaria*) and kombu (*Laminaria*), with the first two commonly used in the preparation of sushi.

However, other types of processed algae are commercialized in Asia such as gim snack (seasoned laver), mareun-gim (dried laver), okazu nori (laver for side dish), yakinori (roasted laver) and zicai tang (laver soup), with South Korea, China and Japan producing almost 100% of the total global quantity in 2017 [2]—lavers are a red seaweed species mainly from the genera *Porphyra* and *Pyropia*.

The ancient use of algae dates back to before the domestication of most common animals and plants. For example, the discovery of remnants of eleven species of algae, including partially burned or squashed fragments on stone tools in South Chile, shows that human exploitation and consumption began at least 14,600 years ago [3].

Currently, in several western countries, there is an increasing acceptance of this type of food, not only in vegan and vegetarian diets, often based on the belief that both better health and disease prevention can be achieved using these “natural products” regularly, with emphasis on their intrinsic composition and abundance of several nutrients such as iodine, calcium, iron, potassium or the presence of carbohydrates, proteins, antioxidants and fiber, among others. Nevertheless, the variability of nutritional composition is hugely dependent on the geographical location and the season of collection, beyond the differences within the species, thus decisively influencing their nutritional value [4].

Additionally, studies in drug research have indicated algae as an interesting source to provide novel drugs to treat several diseases, as shown by the commercial application of the most explored chemicals from algae such as terpenes, phenols, quinones, alkaloids, lipids and other related metabolites [5]. When examining the effects of consuming *Chlamydomonas reinhardtii,* it was observed that algae improve human gastrointestinal issues associated with irritable bowel syndrome (IBS) such as diarrhea, gas and bloating [6].

Within the variety of bioactive compounds of brown seaweeds, phloroglucinol, fucoxanthin and fucoidan are among the most abundant, also exhibiting anticancer activity. Antioxidant activity, inhibition of cell proliferation, induction of cell death and suppression of both metastasis and angiogenesis are some of the anticancer mechanisms recognized [7]. In the same context, it was concluded that fucoidan isolated from *Laminaria japonica* exhibited significantly higher superoxide radical scavenging activities as compared to BHA, BHT, and α-tocopherol [8], thus suggesting that fucoidan is an excellent natural antioxidant and has great potential for preventing free-radical-mediated diseases.

Chemical analysis and FTIR spectra revealed the presence of a predominant iota-carrageenan in *Solieria chordalis* (Rhodophyceae) extracted by conventional and microwave-assisted methods. Evaluation of the antiviral activity of iota-carrageenan against HSV-1 (Herpes simplex virus Type 1) showed strong antiviral activity comparable to that verified by the use of the reference drug acyclovir, when carrageenan was extracted by a microwave-assisted method [9].

Despite the huge amount of data emphasizing the importance of natural compounds extracted from algae, several studies also point out the presence of several contaminants in algae-based supplements [10,11,12], which can invalidate the healthy benefits and preventive effects on humans.

Food supplements are concentrated sources of nutrients (i.e., mineral and vitamins) or other substances with a nutritional or physiological effect that are marketed in “dose” form (e.g., pills, tablets, capsules, liquids in measured doses). These are intended mainly to correct nutritional imbalances (https://www.efsa.europa.eu/en/topics/topic/food-supplements, accessed on 12 September 2021). For example, some seaweeds contain concentrations about ten times higher than those found in traditional vegetables, such as iron in *Himanthalia elongata* (Sea spaghetti) in comparison with that of *Lens esculenta* (lentils) or in the case of calcium present in *Undaria pinnatifida* (Wakame) and *Chondrus crispus* (Irish Moss) in comparison with milk [13].

Similarly, supplements based on terrestrial plants can contain high levels of harmful elements such as arsenic and lead, which can exacerbate the risk to public health when the recommended daily intake by the manufacturer is simultaneously high [14]. Furthermore, the contamination of human foodstuff with arsenic, copper, lead and even zinc is a common finding worldwide [15,16,17] with unexpected long-term consequences regarding public health, beyond the accumulation in non-edible species and effects at cellular levels with changes in metabolic pathways [18,19,20,21].

For example, As in rice, the world’s most important food crop, is a reason for concern in Asian countries where As-contaminated groundwater has been used not only for drinking and cooking purposes but also for rice cultivation, which leads to very high concentrations in the rice grain mainly in its inorganic form, which is the most toxic for humans [22].

Regarding multicellular algae, when analyzing 14 species—Rhodophyceae (10), Phaeophyceae (3) and Chlorophyceae (1)—from different coastal zones of Chile, it was observed that total As concentrations varied between 3.0 and 68 mg kg^−1^, while As inorganic concentrations ranged between 0.15 and 1.06 mg kg^−1^ exceeding the limit value (1 mg kg^−1^) established by the regulations of some countries [10].

The analysis of cadmium, lead, mercury, copper, zinc, total arsenic and inorganic arsenic in 11 algae-based products commercialized in Spain for direct human consumption revealed that *Hizikia fusiforme* contained the highest values of total and inorganic As, exceeding the inorganic As limit in all cases (which would preclude its consumption by humans), whereas most samples exceeded the limits set for Cd in French Legislation [11].

In the same context, a total of 73 samples of edible algae from different origins such as China, Thailand, Taiwan and European coastal waters (Spain and Portugal) commonly sold in the island of Tenerife (Canary Islands, Spain) were analyzed, regarding the presence of different heavy metals [12]. The Asian algae had the highest concentrations of Cd and Pb while the algae from Europe had the highest Hg content. The consumption of 5 g per day of dehydrated Asian wakame algae would account for 22.7% of the tolerable weekly intake of Cd, a high percentage if we take into account the overall load during feeding requirements [12].

Conversely, when analyzing the concentrations of Ni, Zn, Hg, Pt, Mg and Mn in 25 Spirulina products commercialized worldwide for direct human consumption, it was observed that the concentrations do not exceed the present regulation levels, thus can be considered as safe [23].

In Portugal, there is an increasing awareness that algae products are important to human nutrition due to the presence of minerals, proteins, vitamins and fatty acid composition, although the country’s production is scarce. According to FAO data, from the 16,696 wet tonnes of green seaweed produced in 2019 (excluding green microalgae), the Philippines, the Republic of Korea and South Africa are the leading countries with *Caulerpa* spp., *Monostroma nitidum, Codium fragile* and *Ulva* spp., among others, as the predominant species. A small amount of the above-mentioned total value was also mentioned (486 wet tonnes), with Portugal being responsible for 7.2%, Spain with 0.2% and Vietnam with 92.6%, although in this case, there is no indication regarding the type of species produced [24].

Algae research and development and industrial activities are growing in Portugal. Established players include Algaplus, the leading producer of macroalgae in Portugal, mostly for food use, and Iberagar, the leading company in the production of agar [25]. The microalgae players include A4F (Algae for future), AllMicroaelgae, Buggypower and Necton. A large research center and pilot site (a total of 14 ha) named ALGATEC—Eco Business Park is currently in development near Lisbon [25].

Portugal’s imports of seaweeds and other algae fit for human consumption amounted to 498,000 USD in 2012 increasing to 921,000 USD in 2014 and decreasing to 794,000 USD in 2016 [26], clearly indicating a production deficit, which is extensive in the production of agar and carrageenan since the imports in 2016 reached 241,000 USD and 1.78 million USD, respectively. Additionally huge is the deficit regarding the import of seaweeds and other algae not fit for human consumption in 2016—2.32 million USD [26].

Considering the importance of supplements worldwide, this study aimed to evaluate the elemental composition of fifteen different algae-based supplements by µ-EDXRF (fourteen of them in the form of ground powder and one (North Atlantic Kelp) in a pill format), with different origins, taking into account the daily intake recommended by the manufacturer vis a vis the Daily Reference Intake (DDI) of mineral for adults indicated by the European Food Safety Authority (EFSA).

## 2. Results

### 2.1. Elemental Composition

‘Kelp’ is the common name for a group of large brown algae (Phaeophyceae), sometimes erroneously used to describe large brown algae from different orders [27]. In the current study, one of the samples (KelpBio) is based on *Ascophyllum nodosum* (Order Fucales), while the other three samples do not indicate the species used in the formulation, although the term kelp commonly refers to members of the order Laminariales. A particular sample, so-called Kombu, is easily identified because it is well known that this name in Japan is related to *Laminaria* algae.

Interesting differences were observed among the samples particularly related to the presence of both macro and micronutrients, beyond the presence of As. The North Atlantic Kelp (KelpNA) has 3.32% of Ca while in the Kelps from Canada (KelpC), Ireland (KelpI) and from Norway—organic cultivation (KelpBio)—the concentrations of Ca range between 0.822% and 0.997%. The Kelp from Japan (KelpJ) contained only 0.519% μg/g Ca (Table 1), showing that apart from the different origin and cultivation methods, they could indeed be from different species although sold under the broad label ‘Kelp’, or belonging to the same species, which is not indicated in the label.

Regarding K, the concentrations in (KelpC), (KelpI) and (KelpBio) are close, while KelpJ exhibited the highest concentration at 22,600 μg/g and the North Atlantic Kelp the lowest one at 2810 μg/g. The distribution of S in algae samples presents high variability with a maximum for KelpI (14,400 μg/g) and a minimum for KelpNA (3000 μg/g). The element was not detected in KelpBio and KelpJ (Table 1).

Kombu (KelpJ) is by far the best contributor for iodine with 2270 μg/g, while KelpNA, KelpBio, KelpI and KelpC presented 860, 550, 490 and 160 μg/g, respectively. Cu levels were generally low (<4.0 μg/g), except the concentration observed for KelpJ was 11.0 μg/g, while KelpBio is the only seaweed containing Ni with a concentration of 6 μg/g. The highest Fe concentration was observed in KelpC with 404 μg/g while the lowest one was found in Kombu with 29 μg/g, thus showing great variability (Table 1).

The levels of Mn in KelpC, KelpBio and KelpI are close to 20 μg/g. Regarding Zn, the levels were below <10 μg/g in KelpNA and Kombu, whereas the other samples range between 28 and 36 μg/g (Table 1).

Strontium was observed in whole samples, with the highest levels (near or above 600 μg/g) being observed in KelpC, KelpBio and KelpI (*p* < 0.05); KelpJ presents a concentration of 362 μg/g while KelpNA shows a value of 83 μg/g (Table 1). The presence of Ti was common in all the samples except KelpC, with the highest concentration verified in Kombu (86 μg/g). The presence of As is also common in all the samples despite great variability—60 μg/g in Kombu and 8.7 μg/g in KelpNA (Table 1).

When analyzing the macronutrient levels observed in Wakame (*Undaria pinnatifida*), Arame (*Eisenia bisyclis*), Sea spaghetti (*Himanthalia elongata*), Hijiki (*Hizikia fusiformis*) and Agar-agar (*Gracilaria verrucosa*), it was observed that Arame has approximately 1% Ca, while Wakame and Sea spaghetti exhibited similar values between 0.69 and 0.74% and Agar and Hijiki the same concentration i.e., 0.38% (Table 2). K is abundant in Sea spaghetti and Wakame with concentrations around 6.0%, while Hijiki and Arame samples do not reach 1.0%. Iodine was only detected in Hijiki and Arame with mean values of 250 and 400 μg/g, respectively (Table 2).

The levels of Cu range between 4.0 μg/g (Wakame) and 13.4 (Agar), while the levels of Fe range between 29 (Sea spaghetti) and 70 μg/g (Hijiki). Very low concentrations of Ni were observed in two samples only, with a similar situation occurring for Mn, although in the last case, the levels of Hijiki and Sea spaghetti double those levels measured in Agar (11.8 μg/g)—Table 2.

Zn average values are generally around 30 μg/g, except Wakame with 7 μg/g and Agar with 21 μg/g. The levels of Sr exhibited great variability ranging between 9.7 μg/g in Agar and 2220 μg/g in the Arame algae (Table 2). The concentrations of Sr in Spirulina and *Chlorella* are not significantly different at the 0.05 significance level.

In four cases, Pb was present with concentrations ranging between 9.7 and 19 μg/g, i.e., in Sea spaghetti and Arame, respectively. As was found in all the samples with very high levels in the majority of the cases. In fact, Hijiki and Arame contained 66 and 60 μg/g, respectively, while the mean values found in Wakame and Sea spaghetti were 47.0 and 36 μg/g, respectively (Table 2).

Regarding the elemental composition of Nori samples, *Ulva*, the unicellular algae *Chlorella* and Cyanobacteria, it was observed that K predominates with concentrations reaching a maximum of 2.9% in NoriSK, while Ca seldom exceeds 0.5%. Sulphur exhibits an intermediate behavior ranging between 0.52% and 2.18%, being absent from Spirulina (Table 3).

The highest concentrations of Cu were observed in Nori from both South Korea and China, the same as for Zn. Regarding Fe and Mn, the highest levels were measured in *Ulva* and *Chlorella*, and in a single case, Mn was not detected in Spirulina samples (Table 3).

The levels of Sr exhibited great variability with concentrations close to 11 μg/g as in the case of *Chlorella* and Spirulina, while *Ulva*, for example, contains an average of 73 μg/g. The Nori samples are quite distinct with the NoriSK containing 52 μg/g against 33 μg/g from NoriCHN (Table 3).

As exhibits a ubiquitous presence with levels near 20 μg/g in two cases, as detected in Nori samples from South Korea and China. The lowest As concentration was observed in Spirulina with 4.0 μg/g. Ti was only present in *Ulva* sp. and Spirulina samples with concentrations ranging between 24.8 and 27.0 μg/g (Table 3).

### 2.2. Principal Component Analysis (PCA)

In order to integrate the analysis of the minerals in the different food supplements, a Principal Component Analysis (PCA) was carried out and seven attributes (elements) were used, namely Ca, K, Cu, Fe, Zn, Sr and As. The first three main components explained 74.27%: 34.6% for the first principal component (PC1), 21.3% for the second (PC2) and 18.4% for the third (PC3). Only these components were considered significant since only these components showed a correlation with an absolute value >7 with one or more original variables. Thus, the first three components were defined as the main components (Table 4).

In order to understand the relative importance of each attribute in relation to each of the first three main components, the correlation coefficients between the attributes (original parameters) and the main components were determined (Table 5).

**Figure 1:**
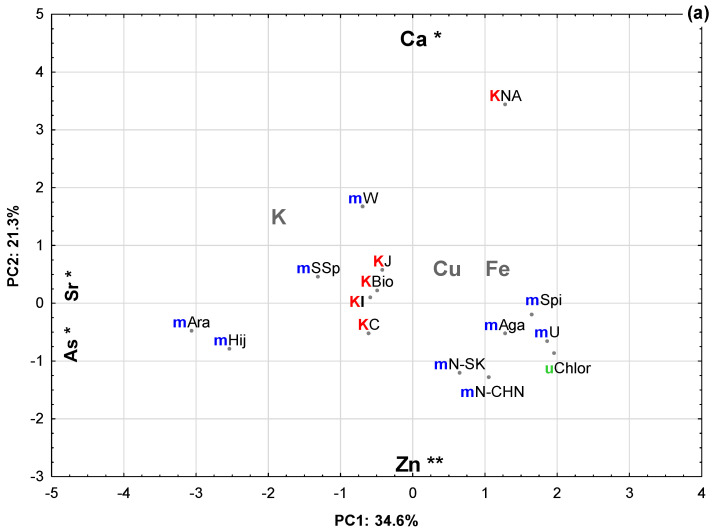

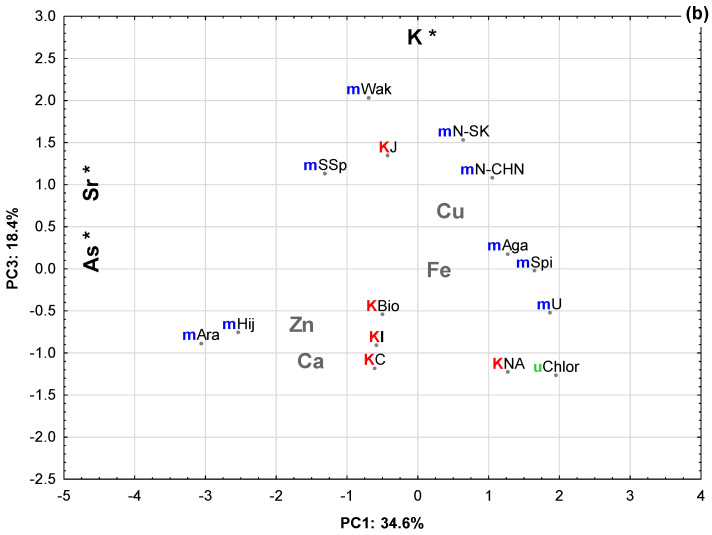
Principal component analysis projection of samples: (**a**) PC1 vs. PC2 and (**b**) PC1 vs. PC3. The most important variables for the definition of the two components are shown in each axis, indicating the direction in which each element grows. KC, KBio, KI, KNA and KJ, represents samples of Kelps from different origins. The designation different multicellular organisms are abbreviated (example mHij refers to Hijiki) and uChlor refers to unicellular *Chlorella*. ** moderately significant correlation values between the element and the PC; * strongly significant correlation values between the element and the PC.

PC1 was explained by the minerals Sr and As (with negative correlation values), PC2 showed a negative correlation with Zn and a positive correlation with Ca, and PC3 showed a positive correlation with Cu. In Figure 1, the projection of the samples in the two main plans can be observed, constituted by the first three components, namely PC1 × PC2 and PC1 × PC3, associated with the approximate projection of the attributes in each plane.

### 2.3. Discussion

Due to its high accumulation capacity, seaweed can be an important source of increased exposure to potentially harmful elements, such as Cd, Hg, Pb and inorganic As (iAs), or even I [30], an essential component of the thyroid hormones thyroxine and triiodothyronine.

The concentration of Cu in KelpJ is in full agreement with the level referred to for *Laminaria* sp. with the same origin, although the levels of Ca, Fe, Sr and Zn were approximately two times higher than our similar values [31]. The same authors point out huge differences among the different elements regarding the localities where the samples were collected. For example, while the levels of Cu were similar regardless of the origin (France, Spain, Korea and Japan), the levels of Fe in *Laminaria* from Korea were approximately seven-fold higher than the levels verified in Japanese samples. Moreover, the Mn concentration in Korean samples is fourteen times higher than the concentration values of similar samples from Japan, where a value of 3.0 μg/g [31] was observed. In our case, Mn was not detected in samples from Japan and North Atlantic, although it was present in other Kelp samples.

The presence of iodine in all Kelp samples means that this type of foodstuff is a good source of I but, in parallel, might contribute to excess intake in the human body. Iodine can cause dysfunction of the thyroid gland at high levels of exposure. Thus, in 2014, EFSA’s Scientific Committee on Food suggested an adequate intake (AI) for adults of 150 μg iodine/day [32], although in the case of pregnant women, a concentration of 200 μg iodine/day had been proposed.

According to the label, North Atlantic Kelp has 200 μg I in each pill, with a recommended intake of a pill per day. Given that the pill’s weight is around 143 ± 1 mg, this corresponds to a concentration of around 1400 μg/g. Our results show that the I concentration in that sample is 860 μg/g, which is almost 40% lower than the labeled value. Given the recommended intake of one pill by the supplier, the legal amounts of I are not exceeded. This pill also contains other ingredients, such as Microcrystalline Cellulose, Dicalcium Phosphate, Vegetable Stearic Acid, Vegetable Cellulose and Vegetable Magnesium Stearate.

As is commonly found in different algae, mainly in organic forms, the less toxic species [33,34]. In Khan et al. [33], all the edible seaweeds—laver (*Porphyra tenera*), seatangle (*Laminaria japonica*), sea mustard (*Undaria pinnatifida*), hijiki (*Hizikia fusiforme*) and gulf weed (*Sargassum fulvellum*)—purchased from supermarkets in South Korea exhibited different levels of As, with the highest concentrations observed in hijiki and gulf weed with 4.49 and 6.48 μg/g (on a fresh weight basis), respectively. These last two algae were also those where inorganic As was found at levels of 2.35 and 5.34 μg/g, respectively [33], thus indicating that the remaining seaweeds contained organic As only.

In the paper of Rose et al. [34], concerning the total and inorganic forms of As in 31 samples covering five seaweed species available for retail sale for consumption (samples were purchased as a dried product—nine from Hijiki, three from Arame, five from Wakame and seven from Nori and Kombu), it was observed that As was detected in all samples with total As concentrations ranging from 18 to 124 μg/g. Inorganic As was only found in the nine samples of hijiki seaweed, at concentrations ranging from 67 to 96 mg/kg. The other seaweeds were all found to contain <0.3 μg/g inorganic As, which was the limit of detection for the method used [34].

The above-mentioned findings indicate that some species, particularly hijiki seaweed must be used with great caution due to the abnormal levels of inorganic As (which is more toxic than organic As compounds), with As(III) more harmful to human health than As (V) [35]. Furthermore, it was already observed that the inorganic As content of the Phaeophyceae *Laminaria digitata* and *Ascophyllum nodosum*, using inductively coupled plasma mass spectrometry and high-performance liquid chromatography, varies significantly according to the studied species. The total As ranged from 36 to 131 mg kg^−1^ dry weight (DW) in *L.* digitata, and from 38 to 111 mg kg^−1^ DW in *A*. *nodosum*. However, in *A. nodosum*, the inorganic As concentration was less than 1% of the total As content, while in *L. digitata*, it ranged between 2.2 and 87 mg kg^−1^, increasing through the thallus from the stipe to the decaying distal blades, reaching more than 50% of the total As [36].

For human consumption, there are no EC regulatory limits set for either total or inorganic arsenic, although Australia and New Zealand [37] have established maximum levels of inorganic As of 1 mg kg^−1^ for seaweed and mollusks used in human feed. Using Caco-2 cells as an in vitro model, it was observed that continuous exposure (up to 21 days) to inorganic As at concentrations that are common in contaminated drinking water and some foods affect the intestinal epithelium structure, such as causing a loss of microvilli as well as compromising epithelial repair mechanisms [38].

Furthermore, the CONTAM Panel identified a range of benchmark dose lower confidence limit (BMDL01) values between 0.3 and 8 μg/kg body weight per day for cancers of the lung, skin and bladder, as well as skin lesions. The scientific opinion concluded that the estimated dietary exposure to inorganic As for average and high-level consumers in Europe is within the range of the BMDL01 values identified, and that, therefore, there is little to no margin of exposure, and the possibility of risk to some consumers cannot be excluded [39].

Despite these statements, some authors concluded [33], based exclusively on the levels detected, that the edible seaweed species analyzed were safe and their contribution of inorganic As to the daily diet is within the permissible specified limits. This assumption often does not take into account the recommended daily intake by the manufacturer, nor the cumulative effect during the life span of a particular individual.

Extensive work regarding supplements based on terrestrial plants showed that a huge increase in public health risk exists when the recommendations of the manufacturer are followed, even in the presence of low concentrations of As [13]. In this context, the UK Food Standards Agency (FSA) issued advice to consumers to avoid eating hijiki seaweed since its consumption could significantly increase dietary exposure to inorganic As [34].

Strontium is an element with chemical behavior similar to that of Ca, and 90 Sr behaves as a bone-seeking nuclide. Thus, when entering the food chain and, consequently, the human body, it is deposited in bone (the human skeleton consists of about 40% Ca) and teeth [40]. Furthermore, the alginate richness in brown algae potentiates the accumulation of Sr, due to their ion exchange properties, particularly those alginates rich in guluronic acid residues with a great affinity for Sr in the ion exchange reaction Sr-Ca [41], which is in agreement with our findings since the kelp (KelpNA) with the lowest Sr concentration exhibits the highest concentration of Ca, clearly indicating competition in the metabolic uptake pathway.

Commercial fertilizers containing Ti, such as Tytanit and Mg-Titanit, have been used for improving crop production. It seems that Ti helps induce the expression of genes related to Fe acquisition when plants are Fe deficient. When Ti concentration in plants is high, Ti competes with Fe for ligands or proteins [42]. Thus, a dual phenomenon exists (antagonism or synergism) depending on the overall concentrations of both elements.

Due to the low solubility of Ti minerals during rock weathering and pedogenesis, Ti can accumulate in the soil, but not in aquatic environments, particularly seawater. Thus, algae and submersed angiosperms are low in titanium [43], except diatoms, confirming the low levels generally found in the overwhelming majority of samples in the current study.

As stated before, As was found in all the studied samples—Wakame (*Undaria pinnatifida*), Arame (*Eisenia bisyclis*), Sea spaghetti (*Himanthalia elongata*), Hijiki (*Hizikia fusiformis*) and Agar-agar (*Gracilaria verrucosa*). Pb was found in four of them with levels ranging between 9.7 and 19.0 μg/g. This contrasts with the very low levels of Pb observed in the same edible species, where the maximum concentration was 1.28 μg/g—on a dry weight basis [11].

**Figure 2:**
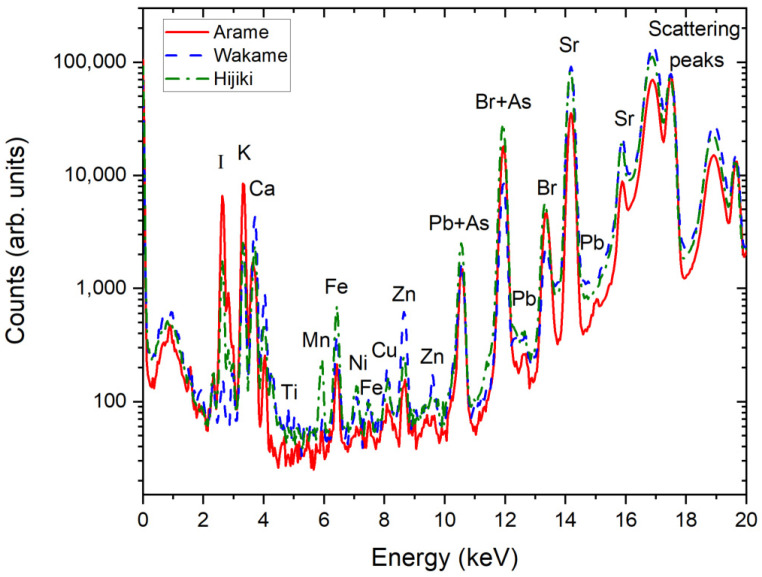
X-ray spectra of one sample of Arame, Wakame and Hijiki. The characteristic peaks of most of the major and trace elements are presented in the graph, where some overlap between Pb, As and Br are evident. The existence of isolated Pb peaks allows for correct quantification of each of these three elements. The two doublets at the right side of the spectra are the Compton and Rayleigh peaks from X-ray scattering in the secondary Mo target.

The X-ray spectra of Figure 2 are elucidative about the elemental composition of one of the samples of Arame, Wakame and Hijiki algae. As can be seen by the graph, although the assessment of Pb and As contents are difficult in samples that also contain Br, as is the case, the presence of other characteristic peaks for Pb allow an unambiguous determination of each elements’ concentration.

The presence of Pb is a reason of concern since the concentrations observed might be amplified by the daily intake recommended by the manufacturer, as in this case the label does not indicate any recommendation, or by the own will of providing house menus regularly with this type of algae, leading to daily intake doses much higher than the average data found in the European population. Adult exposure was estimated at 0.50 μg/kg b.w. (body weight) per day and the mean lifetime dietary exposure was estimated at 0.68 μg/kg b.w. per day in the European population based on middle bound mean Pb occurrence [44].

Pb has been considered a major threat for human health due to the induction of inflammatory cascades in various tissues. It may cause respiratory, neurologic, digestive, cardiovascular and urinary diseases [45]. In humans, Pb is distributed into soft tissues (brain, liver and kidney) and particularly bones, where it accumulates over time. There is no known level of Pb exposure that is considered safe [46]. Young children are particularly vulnerable and can suffer profound and permanent adverse health effects, affecting the development of the brain and nervous system. In adults, it also causes long-term harm, including increased risk of high blood pressure and kidney damage. Pb in bone is released into the blood during pregnancy and becomes a source of exposure to the developing fetus, causing stillbirth, premature birth and low birth weight [46].

Regarding As, great variability within different algae species was observed, with Hijiki reaching a maximum concentration of 147 μg/g total As and 69.5 μg/g of inorganic As [11]. In the current study, the maximum As concentration was also detected in Hijiki with 66.0 μg/g. In *Gracilaria chilensis* harvested from coastal areas of Chile, a total of 7.5 μg/g As was detected, with 0.93 μg/g being inorganic As [10], while in our case, the algae *Gracilaria verrucosa* contained a total As level of 3.9 μg/g.

Specimens of red algae *Gracilaria gracilis* (Gracilariales), harvested from the Central West Portuguese Coast (Buarcos bay), were analyzed in terms of chemical composition including elemental characterization [47]. The concentrations of Ca and Zn in *G. gracilis* are very similar to the levels observed by us in *G.verrucosa* samples, although the levels of Cu and Fe are opposite—Cu levels in *G.verrucosa* are approximately three times higher than similar values found in *G. gracilis*, with the reverse occurring for Fe.

Great variability in Cu and Zn mean values was noted in Wakame, Arame, Sea spaghetti, Hijiki and Agar-agar—Cu ranged between 0.41 and 7.70 μg/g, and Zn between 1.30 and 48.7 μg/g [11]. Another study reported, in Arame and Wakame, much lower variability of Cu and Zn levels, also indicating that the concentrations of Ca, Fe and Pb are too low [48] when compared with our respective results, showcasing huge sample variability, which is mostly related to the origin, development degree of the species, and the seasonality, beyond specific uptake mechanisms.

Regarding the elemental composition of *Chlorella* sp., *Ulva* sp., (Sea lettuce), *Porphyra* sp. (Nori) and the *Cyanobacteria Spirulina* sp., it was observed that As predominates in NoriCHN and NoriSK with approximately 20.0 μg/g, with the lowest level being measured in *Spirulina* sp. with 4.0 μg/g. The algae *Ulva rigida* exhibited an As range of 6.41–7.06 μg/g, while Cu and Zn ranged 3.05–3.15 μg/g and 5.61–6.14 μg/g, respectively [11]. The same authors found a total As concentration ranging 28.9–49.5 μg/g [11] in *Porphyra umbilicalis*, somewhat higher than those observed by us in samples from South Korea and China.

Conversely, the levels of total As in *Porphyra* sp harvested from different coastal areas in France, Spain, South Korea and Japan revealed that As concentrations range between 4.25 μg/g in France and 9.70 μg/g in Japan, on a dry weight basis [49].

The highest levels of Ca, Cu, K, S and Sr were observed in the unicellular algae (*Chlorella*) and in the *Cyanobacteria Spirulina*. The unicellular algae *Chlorella* sp. and *Arthrospira* sp. (known commercially as Spirulina) are currently cultivated for the purpose of production of food supplements, thus the quality and safety of these products is crucial for human health. The evaluation of the elemental composition of 23 supplements registered in the European Union was evaluated [50]. The macronutrient levels in Spirulina supplements decreased in the order of K  >  Ca  >  P  >  Na  >  Mg, while for *Chlorella* supplements, the concentrations decreased in the order of K  >  P  >  Ca  >  Na  >  Mg. Furthermore, a significantly higher content of Ca was observed for Spirulina products while *Chlorella* displayed a greater P level [50]. In our case, we only had data from K and Ca, and the levels of K are always higher than those of Ca.

Regarding micronutrients, the mean values decreased in the following order in Spirulina supplements: Fe  >  Mn  >  Zn  >  Cu  >  Cr  >  Co  >  Mo  =  Se, while in *Chlorella*-based supplements, the order is slightly different—Fe  >  Mn  >  Zn  >  Cu  >  Cr  >  Mo  >  Se  >  Co [50]. In our case, the order for *Chlorella* is identical (Cr, Mo, Se and Co were not identified in either *Chlorella* nor in Spirulina), while in Spirulina, the rank is the following: Fe > Zn > Cu, since Mn is below the detection limit.

Other studies report very low levels of both Mn and Zn in 25 different commercial Spirulina products. The concentrations of Mn and Zn on a dry weight basis are in the range of 0.005–2.248 μg/g and 0.533–6.225 μg/g, respectively, leading the authors to claim that all the Spirulina samples were considered to be safe [23].

In the current study, the multicellular algae *Ulva* sp. and the unicellular algae *Chlorella* sp. are a particular source of Fe with ca. 800 μg/g. In addition, the highest levels of Mn were found in the same algae, but the levels of *Ulva* are three-fold higher than the concentrations of *Chlorella* sp. while the concentrations of Zn are similar.

Titanium, as previously stated, is poorly accumulated in algae and submersed angiosperms (except diatoms) [43], which agrees with our results, although *Ulva lactuca* collected in different coastal areas of Turkey in two consecutive years revealed very high values of Ti with a minimum of 2280 μg/g and a maximum of 10,770 μg/g [51]. The same authors also report very high levels of Fe and Sr in the *Ulva* samples [51], contrasting with our low levels, clearly indicating that the environmental conditions, beyond factors intrinsic to the species, are responsible for the variability observed.

Regarding Principal Component Analysis, three groups can be established depending on the concentrations of As and Sr observed: Those with higher values, Arame and Hijiki; those with lower values of these elements, KelpNA, Spirulina, Agar, *Ulva* sp., NoriSK, NoriCHN and *Chlorella* sp.; and the remaining supplements have intermediate values. As for the amounts of Ca and Zn, KelpNA and Wakame, with high values of Ca and low values of Zn, stand out, as does Nori with low values of Ca and high values of Zn (Figure 1a). Additionally, the high K values in the Sea spaghetti, Wakame, NoriSK and NoriCHN samples can be emphasized (Figure 1b) as well as a very noticeable similarity of K levels in all Kelps (except KelpNA).

In the majority of the cases, there are no recommended daily intakes by the manufacturer, as although the median of cooked Hijiki in Japan was 5.5 g/day [52], it is well known that this seaweed contains high values of total As and considerable concentrations of inorganic As [10,11]. The concentration of inorganic As in Hijiki range between 83 and 88 mg kg-1 while the total As ranges between 115 and 141 mg kg-1. These abnormal concentrations of the inorganic form lead the authors to conclude that daily consumption of 1.7 g of the product would reach the Provisional Tolerable Weekly Intake recommended by the WHO for an average body weight of 68 kg [53], thus the free sale of these types of products must be carefully monitored.

Regarding *Chlorella* and Spirulina, the minimum recommended daily intake is 10 pills per day for adults (each pill weight 0.4 g), which did not constitute a risk despite the increment of total As by a factor four. The presence of Pb in Sea spaghetti, Arame, Hijiki and Wakame caused a considerable risk to public health vis a vis possible ingestion of a daily average of 5 g (dry weight), although other authors point out Pb levels in Arame, Hijiki and Wakame <1 mg kg^−1^ [53], thus indicating that the same species may accumulate highly variable concentrations that do not help the consumer, especially when surveillance is weak or absent.

In our case, the Pb levels are far above the mean levels observed for dietetic products—4.3 mg kg^−1^ with an overall median across all categories of 0.0214 mg kg^−1^ [37], thus indicating these particular samples must be avoided for human consumption. Furthermore, Kelp samples in general, beyond Hijiki and Arame, exhibited very high levels of iodine, in some cases much higher than the adequate intake, which is 150 μg/day for adults according to EFSA [32].

If we consider the samples where there is a clear indication in the label of the daily intake, regarding only the essential elements to human nutrition, such as those observed in the current study (Ca, Cu, Fe, I, K, Mn and Zn), it can be derived that the daily ingestion of 0.7g of KelpBio or KelpI will provide 385 and 343 μ iodine, respectively (Table 6), clearly above the value defined by EFSA as previously discussed, which at its upper limit is 200 μ per day for pregnant women. Regarding the other elements, only Fe deserves attention, in the case of *Chlorella* and Spirulina consumption. In fact, the intake of these products will provide 41% and 16.5%, respectively, of the iron load needed on a daily basis. However, as recently noted, the very high levels of Fe observed in these species obtained from the Slovenian market [54], and particularly Hawaiian *Spirulina pacifica* samples (3.29 mg g^−1^), are probably related to the medium used for cultivation.

The daily intake values take into account the recommended daily intake by the manufacturer. Daily reference intakes for adults expressed as mg or μg established by EU [55] are within brackets.

## 3. Materials and Methods

### 3.1. XRF Preparation and Analysis

All the food supplements are sold freely and are commonly seen in natural product stores. The selected supplements were provided by one of the largest retailers of food supplements in Portugal and reflect some of the most sold products in the country.

According to Portuguese regulations, food supplements must contain a clear definition of the recommended daily dose on the label, which was verified only in six cases, namely all the Kelp samples, except KelpJ or Kombu, and in Spirulina and *Chlorella* products. Thus, the remaining samples are foodstuffs, although this word is a large umbrella that includes drinks, food supplements and any type of food.

Apart from the North Atlantic Kelp (KelpNA), which was in pill format and was then ground, the remaining samples were in powder form, thus all the data are expressed on a dry weight basis. The Recommended Daily Dose (RDD) by the supplier was only observed in six samples with the following recommendations for adults: 0.7 g for KelpBio, KelpC and KelpI, while for Kelp NA a pill per day is suggested, whereby each pill weighs 143 mg. The RDD for Spirulina and *Chlorella* samples is 7.0 g for adults. Batch references of these supplements are omitted for reasons of possible conflict of interest. All the samples were collected from the supplier already grounded and in three individual sealed plastic bags from each species. The powder was pressed for 2 min under 10 t in order to make a cylindrical pellet with a diameter of 20.0 ± 0.5 mm and a thickness of 1.0 ± 0.5 mm and a minimum of three pellets for each sample were made to reduce the error analysis. This pellet was then glued onto a mylar sheet in a plastic frame and placed directly onto the X-ray beam for analysis.

All of the energy dispersive X-ray spectra were acquired with the use of a tri-axial setup, which lowers the detection limits of conventional XRF spectrometers through the polarization of the incident beam at a secondary target [56,57].

The X-ray tube is a Philips PW1400 with a W anode, capable of delivering 100 KV and 80 mA, which is water cooled. The polarizing secondary target is a 99.999% pure Mo disc, and a filter placed before the secondary target absorbs the low energy radiation emitted by the tube. This radiation only contributes to the background by scattering in the secondary target and in the sample. The characteristic radiation of the elements present in the samples is detected by a Si (Li) detector, a 50 mm^2^ active area, an 8 µm beryllium window and 135 eV resolution at 6.4 keV cooled by liquid nitrogen. Two collimators of silver are placed in front of the detector in order to restrict its effective area by excluding regions close to the edges. The acquisition time of each spectrum was 1000 s and the operating conditions of the X-ray tube were 50 kV, 20 mA. Data were stored in a PC computer with a NUCLEUS PCA card. The spectra were evaluated using the fundamental parameters method.

Detection limits, similar to those referred in references [57,58] were the following: As = 3 mg kg^−1^; Ca = 105 mg kg^−1^; Cu = 3 mg kg^−1^; Fe = 6 mg kg^−1^; I = 30 mg kg^−1^; K = 180 mg kg^−1^; Mn = 9 mg kg^−1^; Ni = 3 mg kg^−1^; Pb = 8 mg kg^−1^; Sr = 3 mg kg^−1^; Ti = 15 mg kg^−1^; Zn = 3 mg kg^−1^. Plant reference materials were used for data validation: Orchard leaves (NBS 1571) and poplar leaves (GBW 07604); the recovery values ranged between 93% and 113%.

### 3.2. Principal Component Analysis

Principal Component Analysis is a dimension-reduction tool that can be used to reduce a large set of variables to a small set that still contains most of the information from the large set. In this context, PCA was applied to observe any possible clusters within the different seaweeds as well as unicellular organisms such as *Chlorella* sp. and Spirulina and *Ulva* sp., a multicellular green alga.

### 3.3. Statistical Analysis and Control Assurance

Statistical analysis of the data was performed with the SPSS Statistics 18 program, through an analysis of variance (ANOVA) and the F-test. A value of *p* ≤ 0.05 was considered to be significant. All analyses were made in triplicate. Analytical accuracy was verified using replicate determinations and standard reference materials, as referred above, with percentages of recovery ranging between 93% and 113%.

## 4. Conclusions

The observed values of both the macro and micronutrients present in the samples studied in this work allow us to conclude that algae-based food supplements have very interesting nutritional value. Nevertheless, the presence of toxic elements in concentrations that lead to daily intakes of these elements that exceed E.U. regulations is a concern that might hinder more widespread consumption of these products. Regarding macronutrients, our findings are in accordance with most studies that focus on the elemental concentration of these nutrients with a wide set of analytical techniques. The presence of trace amounts of toxic elements is highly dependent on the provenance of the raw material of the supplements, which should represent the environmental conditions at the place of origin, rather than the external contamination arising from processing.

Given the very high concentrations of Pb, As and even I in some samples, and the absence of recommendations regarding both the presence of this type of contaminant and the daily intake dose, an increasing public health risk exists if consumers adopt a strategy of replacing home natural food sources with products such as algae or terrestrial plant-based supplements. Consumers must not approach these natural products as a panacea for nutritional imbalances. Instead, there is an urgency to demand that national health authorities adopt clear and effective strategies of control quality with the aim of safeguarding the public health.

Although this particular study raises some concerns regarding the quality and safety of algae-based supplements sold in Portugal, the number of samples and their sources are not completely representative of the market. A much larger screening of all of the commercialized algae-based supplements is warranted in order to provide the national health authorities with a complete picture regarding possible legislation issues with these food supplements.

## Figures and Tables

**Table 1:** Elemental composition of Kelp from different origins.

Element	KelpC	KelpBio	KelpI	KelpNA	KelpJ
Ca	8220 ± 130b	8700 ± 100b	9970 ± 450b	33,200 ± 3500a	5190 ± 350c
K	15,100 ± 230b	14,760 ± 340b	17,000 ± 400b	2810 ± 270c	22,600 ± 1900a
S	10,500 ± 900bc	BDL	14,400 ± 1800a	3000 ± 1000c	BDL
I	160 ± 50d	550 ± 70c	490 ± 50c	860 ± 160b	2270 ± 90a
Cu	4.0 ± 1.0b	4.0 ± 0.8b	3.6 ± 0.4b	3.0 ± 0.7b	11.0 ± 0.6a
Fe	404 ± 5a	116 ± 16b	381 ± 14a	71 ± 5c	29 ± 2d
Mn	19.1 ± 1.7a	16.9 ± 2.7a	20.0 ± 1.8a	BDL	BDL
Ni	BDL	6 ± 3a	BDL	BDL	BDL
Zn	36 ± 2a	28 ± 1b	28 ± 1b	7 ± 1c	9 ± 1c
Sr	640 ± 39a	582 ± 31a	690 ± 50a	83 ± 7c	362 ± 22b
Ti	BDL	25 ± 7b	34 ± 20b	27 ± 6b	86 ± 8a
As	34.0 ± 0.6c	29 ± 2d	40 ± 1b	8.7 ± 0.2e	60 ± 6a

Average concentrations in the same row, followed by a common letter, are not significantly different at the 0.05 significance level; mean values obtained on a dry weight basis are expressed in μg/g ± standard deviation; *n* = 3; BDL = Below the Detection Limit.

**Table 2:** Elemental composition of different multicellular algae.

Element	Hijiki	Agar	Arame	Sea Spaghetti	Wakame
Ca	3900 ± 2700c	3830 ± 220c	9900 ± 1200a	7400 ± 400b	6960± 190b
K	6260 ± 430c	120 ± 23d	9800 ± 1000b	57,000 ± 5000a	62,000 ± 5000a
Cu	5.6 ± 0.9c	13.4 ± 2.0a	9.0 ± 1.7ab	5.0 ± 1.1c	4.0 ± 0.8c
Fe	70 ± 10a	34 ± 3bc	58 ± 22ab	29 ± 3c	43 ± 2b
Mn	27.8 ± 3.5a	11.8 ± 2.0b	BDL	22.0 ± 2.4a	BDL
Ni	BDL	BDL	3.5 ± 0.1	4.0 ± 0.2	BDL
Zn	36 ± 2a	21 ± 3b	35 ± 17ab	30 ± 1a	7 ± 1c
I	250 ± 60b	BDL	400 ± 29a	BDL	BDL
Sr	1420 ± 100b	9.7 ± 1.2d	2220 ± 230a	690 ± 80c	560 ± 70c
As	66 ± 17a	3.9 ± 0.3d	60 ± 18ab	36 ± 2c	47.0 ± 0.6b
Pb	13 ± 1a	BDL	19 ± 11a	9.7± 0.5a	10.0 ± 0.8a

Average concentrations in the same row, followed by a common letter, are not significantly different at the 0.05 significance level; mean values obtained on a dry weight basis are expressed in μg/g ± standard deviation; *n* = 3; BDL = Below the Detection Limit.

**Table 3:** Elemental composition of different unicellular (*Chlorella*) and multicellular algae, (Sea lettuce—*Ulva* sp.; Nori—*Porphyra* sp.) and the *Cyanobacteria Spirulina* sp.

	*Ulva*	NoriSK	NoriCHN	*Chlorella*	Spirulina
Ca	5300 ± 500a	3030 ± 240b	4680 ± 200a	1400 ± 80c	970 ± 420c
K	18,600 ± 1500b	29,100 ± 1800a	13,800 ± 440c	7420 ± 340d	11,220 ± 620c
S	21,800 ± 2000a	7000 ± 4800b	17,700 ± 1000a	5200 ± 900b	BDL
Cu	10.0 ± 1.3b	21 ± 3a	23 ± 5a	4.6 ± 0.4c	7 ± 3b
Fe	792 ± 24a	153 ± 62c	180 ± 80c	826 ± 33a	330 ± 41b
Mn	151 ± 12a	24.9 ± 2.6c	24.4 ± 1.9c	49 ± 3b	BDL
Zn	18 ± 6ab	26 ± 2a	24 ± 3a	20 ± 1b	14 ± 6bc
Sr	73 ± 8a	52 ± 5b	33 ± 3c	11.0 ± 1.2d	12.0 ± 0.9d
Ti	24.8 ± 3.1a	BDL	BDL	BDL	27.0 ± 2.1a
As	10.8 ± 0.4b	22.7 ± 1.1a	19 ± 3a	7.0 ± 0.8c	4.0 ± 1.6cd

Average concentrations in the same row, followed by a common letter, are not significantly different (*p* ≤ 0.05); mean values obtained on a dry weight basis are expressed in μg/g ± standard deviation; *n* = 3; BDL = Below the Detection Limit.

**Table 4:** Eigenvalues of the correlation matrix and related statistics. Percentage of variance for each component (initial eigenvalues).

	Eigenvalue	Total Variance (%)	Cumulative Eigenvalue	Cumulative (%)
1	2.42	34.57	2.42	34.57
2	1.49	21.31	3.91	55.88
3	1.29	18.40	5.20	74.27
4	1.01	14.44	6.21	88.71
5	0.40	5.75	6.61	94.47
6	0.27	3.89	6.89	98.36
7	0.11	1.64	7	100

**Table 5:** Correlation coefficients between attributes (initial variables) and PC1 and PC2.

	Components		
Attribute	PC1	PC2	PC3
Ca	−0.02	0.82 *	−0.33
K	−0.24	0.22	0.71 *
Cu	0.26	−0.53	0.51
Fe	0.53	−0.35	−0.46
Zn	−0.57	−0.60 **	−0.34
Sr	−0.93 *	−0.07	−0.24
As	−0.91 *	0.01	0.16

* Values considered strongly correlated with PC (|r| > 0.7); ** values considered moderately correlated with PC (0.6 < |r| < 0.7); following the classification used previously [28,29].

**Table 6:** Daily intakes for particular elements provided by the consumption of Kelp, *Chlorella* and Spirulina.

Studied Species	Ca (800 mg)	Cu (1 mg)	Fe (14 mg)	I (150 μg)	K (2000 mg)	Mn (2 mg)	Zn (10 mg)
KelpC	5.75	0.0028	0.283	112	10.6	0.013	0.025
KelpBio	6.09	0.0028	0.081	385	10.3	0.012	0.020
KelpI	6.98	0.0025	0.266	345	11.9	0.014	0.020
KelpNA	4.74	0.0004	0.010	123	0.40	--	0.001
*Chlorella*	9.80	0.032	5.78	--	51.9	0.34	0.14
Spirulina	6.79	0.049	2.31	--	78.5	--	0.098
